# Interface Adhesion Property and Laser Ablation Performance of GAP-PET Double-Layer Tape with Plasma Treatment

**DOI:** 10.3390/nano12111827

**Published:** 2022-05-26

**Authors:** Sibo Wang, Bangdeng Du, Baoyu Xing, Yanji Hong, Ying Wang, Baosheng Du, Yongzan Zheng, Jifei Ye, Chenglin Li

**Affiliations:** 1 State Key Laboratory of Laser Propulsion & Application, Space Engineering University, Beijing 101416, China; bosiwang1@163.com (S.W.); bddu13s@alum.imr.ac.cn (B.D.); xingbaoyu1984@aliyun.com (B.X.); yingwang8971@163.com (Y.W.); dubaosheng123@126.com (B.D.); yongzanzheng@163.com (Y.Z.); yjf1981@163.com (J.Y.); lichenglin0721@163.com (C.L.); 2 Technical Institute of Physics and Chemistry, Chinese Academy of Sciences, Beijing 100190, China

**Keywords:** laser propulsion plasma treatment, adhesion, ablation efficiency, ablation pit

## Abstract

In the field of laser ablation micro-propulsion, the property of double-layer tape has significant impact on the propulsion performance. In this paper, low temperature plasma was used to treat the surface of polyethylene terephthalate (PET) to improve its adhesion with energetic polymer. The PET surface pre- and post-plasma treatment was characterized by X-ray photoelectron spectroscopy (XPS) and atomic force microscopy (AFM), and the enhancement mechanism of the interface adhesion was discussed. In addition, the ablation performance of the double-layer tape after the plasma treatment was studied. The results showed that the plasma etching effect increased the root mean square roughness of the PET surface from 1.74 nm to 19.10 nm. In addition, after the plasma treatment, the number of C–OH/COOH bonds and O=C–O bonds increased, which also greatly improved the adhesion between the PET and energetic polymers. In the optimization of the ablation performance, the optimal laser pulse width was about 200 μs. The optimal values of the specific impulse (*I*_sp_), impulse coupling coefficient (*C*_m_), and ablation efficiency (*η*) were 390.65 s, 250.82 μN/W, and 48.01%, respectively. The optimization of the adhesion of the double-layer tape and the ablation performance lay the foundation for the engineering application of laser ablation micro-thrusters.

## 1. Introduction

As a new type of aircraft to perform earth orbit or space exploration missions, micro–nano satellites have the advantages of small size, light weight, convenient manufacture, and low cost. Different tasks of micro–nano satellites require accurate, controllable, and continuous thrust output to achieve satellite orbit transfer, attitude adjustment, and fast maneuvering. Therefore, many different kinds of micro–nano satellite micro-propulsion systems have emerged in recent years [1,2,3]. As an alternative, the laser propulsion system has attracted more and more attention due to its characteristics of high specific impulse, low power consumption, small size, and light weight [4,5,6,7].

**Figure 1:**
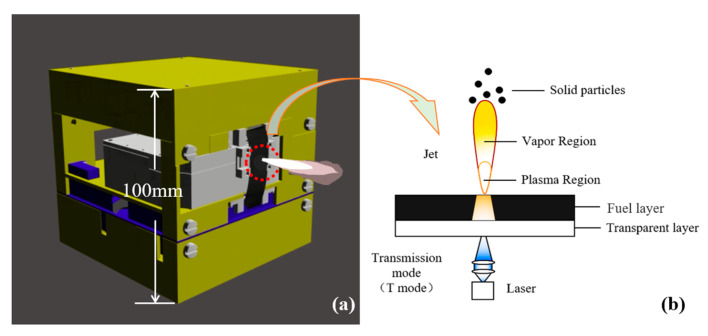
(**a**) The three-dimensional structure of the cube laser ablation micro-thruster; (**b**) The laser ablation principle of transmission mode. Reprinted with permission from [19]. Copyright 2022, IOP Publishing.

The working principle of laser ablation micro-propulsion is that the laser interacts with matter to generate high-temperature and high-pressure plasma or target steam at a certain speed to generate thrust. [8]. Phipps first proposed the concept of the laser ablation micro-thruster in the 1990s [9]. Because of its great potential, it has aroused great interest from researchers all over the world [10,11,12,13,14,15,16,17]. Phipps proposed the model of the double-layer tape [18]. The model of the double-layer tape had also become the main research direction of the laser ablation micro-propulsion technology. A 1U volume double-layer tape laser ablation micro-thruster was designed by our research group. Moreover, its three-dimensional structure and the laser ablation principle of the transmission mode are shown in Figure 1 [19]. In previous studies, researchers paid more attention to the optimization of the fuel layer [20,21,22] and the laser parameters [23]. Moreover, they paid little attention to the interface adhesion property between the transparent layer and the fuel layer. However, the previous experimental results showed that the interface adhesion property between the transparent layer and the fuel layer had a great influence on the ablation performance, especially in the continuous ablation process [24,25], because a large amount of heat can be released during laser ablation. Additionally, an ultra-high pressure zone can be formed inside the ablation pit, which can cause the separation of the double-layer tape.

Therefore, this paper proposed a method to enhance the adhesion of the double-layer tape by the plasma treatment [26]. The surface of the PET pre- and post-plasma treatment was characterized by X-ray photoelectron spectroscopy (XPS) [27] and atomic force microscopy (AFM) [28]. Moreover, the enhancement mechanism of the interfacial adhesion was analyzed. Finally, the ablation performance of the tape after the plasma treatment was optimized and studied by combining the characteristics of the ablation pit profile photographed by scanning electron microscope (SEM) and the laser ablation performance.

## 2. Measurement Methods and Experimental Equipment

### 2.1. Propulsion Parameters

The propulsion parameters are crucial for evaluating the propulsion performances of the micro-thrusters. In laser micro-propulsion, the *C*_m_ (μN/W), named the impulse coupling coefficient, is a vital parameter for evaluating the performance [29]. It is defined as the ratio of the laser-induced single-pulse impulse *I* (kg·m/s) and the incident laser energy *W* (J):

(1) $$C_{m}=\frac{I}{W}$$

In ablation mode, the concept of the specific ablation energy *Q* (J/kg) is defined as the laser energy consumed by the ablation of a unit mass of working matter:

(2) $$Q=\frac{W}{\Delta m}$$

where Δ*m* (kg) is the ablation mass. According to the principle of the conservation of momentum, the working material and ablation products in the ejection process satisfy the following relationship:

(3) $$\Delta mv_{E}=m\Delta v$$

where *v*_E_ (m/s) is the jet velocity and *m*∆*v* is the momentum generated by the working material during the laser ablation jet process. *C*_m_ (μN/W) and *Q* (J/kg) are parameters that can be experimentally measured. Therefore, the jet velocity *v*_E_ (m/s) can be obtained by the following relationship:

(4) $$v_{E}=C_{m}Q$$

The *I*_sp_ (s) is another vital parameter in laser propulsion. The *I*_sp_ (s), named the specific impulse, is defined as the impulse generated by a unit mass of working matter:

(5) $$I_{\mathrm{sp}}=\frac{I}{\Delta mg}$$

where *g* (N/kg) is the acceleration of gravity. From Equations (1)–(5), the relationship between the *I*_sp_ (s) and the jet velocity can be obtained as

(6) $$I_{\mathrm{sp}}=\frac{v_{E}}{g}$$

The *η* (%), called the ablation efficiency, is defined as the conversion of laser pulse energy to jet energy:

(7) $$\eta =\frac{W_{E}}{W}=\frac{\Delta mv_{E}^{2}}{2W}$$

The relationship between the *η* (%), *C*_m_ (μN/W), and *I*_sp_ (s) is as follows:

(8) $$2\eta =C_{m}v_{E}=C_{m}I_{\mathrm{sp}}g$$

### 2.2. Experimental System and Measurement Method

The laser used in this paper is a miniaturized semiconductor laser which has the characteristics of a small volume and a high power density. The pulse width can be adjusted from 50 μs to 1500 μs. The Rayleigh range is about 200 μm. The output power density of the semiconductor laser was about 5 × 10^6^ W/cm^2^ and the wavelength was 975 nm. The tape was a double-layer tape with 87% glycidyl azide polymer (GAP) + 10% ammonium perchlorate (AP) + 3% C (nano-carbon powder) as the fuel layer material (the GAP and AP were produced by the Institute of Explosives and Propellants, Beijing Institute of Technology) and polyethylene terephthalate (PET) (the PET was produced by SABIC (China) Research & Development Co., Ltd., Shanghai, China) as the 100 μm-thick transparent layer, achieving a laser transmissivity of over 99%. The PET had excellent comprehensive properties, including a high mechanical strength, high temperature resistance, and good flexibility, which was very suitable for the transparent layer material of the double-layer tape in laser ablation propulsion [30].

**Figure 2:**
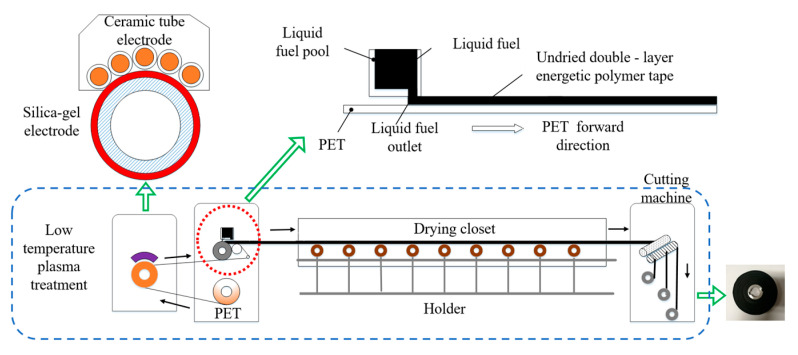
Double-layer tape production system and flow chart.

The production system and process of the double-layer tape are shown in Figure 2. The system was mainly composed of the transmission mechanism, plasma treatment equipment (PG-6000F, produced by Nanjing Suman Plasma Technology Company, Nanjing, China), double-layer tape coating equipment, drying box, and double-layer tape cutting equipment. By adjusting the distance between the blade and the PET, different thicknesses of tape were made. The PET surface was treated with low-temperature plasma in air to improve the adhesion between the PET and GAP. Low-temperature plasma was generated by DBD discharge. Its discharge power range was 100–3000 W. Low-temperature plasma with a power of 1500 W was used to treat the surface of the PET in this paper. The electrode material was corundum ceramic.

**Figure 3:**
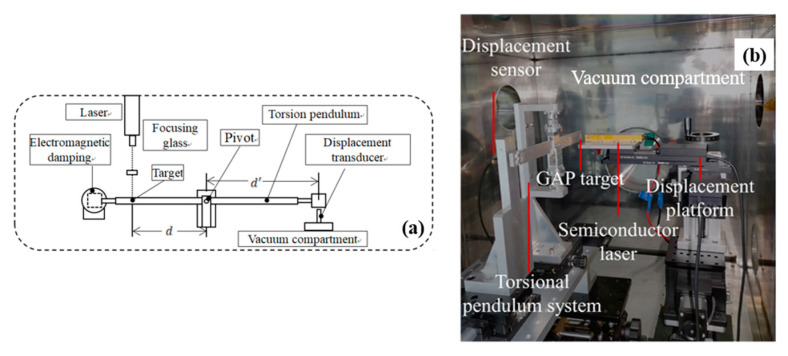
Experimental system. (**a**) Schematic diagram; (**b**) Experimental scene graph. Reprinted with permission from [19]. Copyright 2022, IOP Publishing.

The experimental system is shown in Figure 3a,b [19]. The experimental system includes a semiconductor laser, vacuum chamber, torsion pendulum, displacement sensor, electromagnetic damper, displacement stage controller, and electromagnetic damping controller [31,32]. The vacuum degree of the vacuum chamber can reach 10^−5^ Pa.

The motion of the torsional pendulum system follows the angular momentum theorem, and its equation is:

(9) $$\left\{\begin{array}{ll} J\ddot{\theta }+c\dot{\theta }+k\theta =f(t)d & 0<t<T_{0}\\ J\ddot{\theta }+c\dot{\theta }+k\theta =0 & t>T_{0} \end{array}\right.$$

where *J* is the moment of inertia of the torsional pendulum system, the air damping coefficient is *c*, the stiffness factor of the pivot is *k*, the pendulum deflection angle is *θ*, the deflection angular velocity is $\dot{\theta }$, the angular acceleration is $\ddot{\theta }$, the lever arm length of the external force is *d*, the magnitude of the external force at time *t* is *f(t)*, and the external force action time is *T*_0_.

When $0<t<T_{0}$, the formula is expressed as:

(10) $$\ddot{\theta }+2\xi \omega_{n}\dot{\theta }+\omega_{n}^{2}\theta =f(t)d/J\;\;0\;<\;t<T_{0}\;\omega_{n}=\sqrt{\frac{k}{J}}\;\;\xi =\frac{c}{2\sqrt{kJ}}$$

where ξ is the damping ratio and $\omega_{n}$ is the inherent resonant frequency.

When Θ(s)=L[θ(t)], $\theta_{0}=0$, $\omega_{n}=0$, the impulse is a unit impulse f(t)=δ(t), and the Laplace transform gets:

(11) $$\Theta (s)=(d/J)\frac{1}{s^{2}+2\xi \omega_{n}s+\omega_{n}^{2}}=(d/J)\frac{1}{{\left(s+\xi \omega_{n}\right)}^{2}+\omega_{d}^{2}}$$

where $\omega_{d}=\sqrt{1-\xi^{2}}\omega_{n}$ is the resonant frequency.

After the inverse Laplace transform, the angular change response under the unit impulse is as follows:

(12) $$h(t)=L^{-1}[\Theta (s)]=(d/J)\frac{1}{\omega_{d}}e^{-\xi \omega_{n}t}\sin\omega_{d}t$$

Under the impulse force f(τ)=Iδ(τ) of impulse I, the deflection angle is:

(13) $$\theta (t)=\frac{Id}{J\omega_{d}}e^{-\xi \omega_{n}t}\sin\omega_{d}t$$

### 2.3. Characterization of Double-Layer Tape

**Figure 4:**
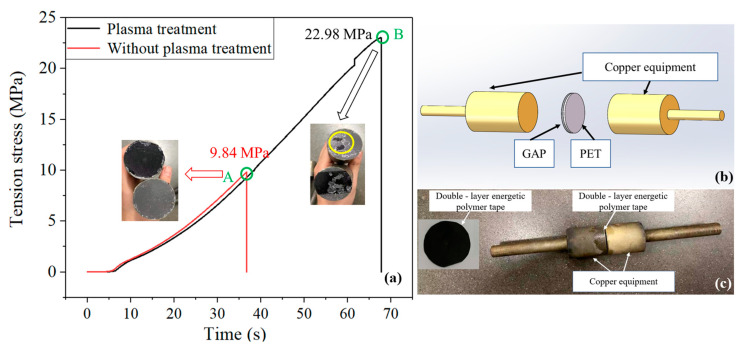
(**a**) Test results of tensile stress. The adhesion between PET and GAP pre- and post-plasma treatment was represented by a red line and black line, respectively. The tensile stress at point A is about 9.84 MPa, and the result of the double-layer band after it was pulled was shown as the arrow at point A. The tensile stress at point B was about 22.98 MPa, and the result of the double-layer band after it was pulled was shown as the arrow at point B. (**b**) 3D schematic diagram of tensile specimen. (**c**) Drawing of tensile specimen.

The specimen used for the tensile test is the double-layer tape with a diameter of 20 mm, and the double-layer tape is constructed by 100 µm-thick PET and 100 µm-thick GAP. Two same tensile dies made of copper were adhered to the two faces of the double-layer tape by using the superglue (Adbest two-component epoxy resin glue made in Shanghai Huayi resin limited company, Shanghai, China), and the tensile specimen is shown in Figure 4b,c. The tensile test was carried out by using a QBD-100 (Jinan Fangyuan Test Instrument Co., LTD, Jinan, China) electronic tensile tester with a tensile speed of 0.5 mm/min.

A Thermo Scientific K-alpha XPS spectrometer (Thermo Fisher Scientific, Waltham, MA, USA) associated with monochromatic X-rays from an Al anode (hν = −1486.6 eV) was adopted to perform the XPS analysis. The energy step size was 0.100 eV, and the pass energy was 20.0 eV. The software “XPS Peak” (4.1; Raymund Kwok) was used for the fitting of the peaks. Moreover, the background was “linear”. The AFM images were adopted via a Bruker Dimension Icon with “Scanasyst-air” AFM tips. The given results of the AFM represent the averages for at least five repeated measurements. The microstructures of the double-layer tape were characterized using the FEI inspect F50 scanning electron microscope (SEM, FEI Company, Hillsboro, OR, USA) with a Quanta 600EDX (Energy Dispersed X-ray) system. The contact angle (CA) of the liquid GAP mixture on the PET was tested by the contact angle instrument (POWEREACH JC2000D3, Shanghai Zhongchen Digital Technology Equipment Co. LTD, Shanghai, China). The given results of CA represent the averages for at least three repeated measurements. The measurement time was completed within 3 s, and the relative error was less than 5%.

## 3. Result and Discussion

### 3.1. Optimization of Double-Layer Tape

The adhesion of the double-layer tape had an important effect on ablation performance. The instantaneous high pressure region generated during laser ablation was easy to cause the separation of the fuel layer and the PET without the plasma treatment. In order to solve the problem, low-temperature plasma with a power of 1500 W was used to treat the surface of the PET in this paper, and the treatment time was about 100 s. The tensile testing machine was used to test the adhesion between the PET and GAP pre- and post-plasma treatment. The PET surface pre- and post-plasma treatment was characterized. The strengthening mechanism of the adhesion was analyzed.

#### 3.1.1. Adhesion Test and Contact Angle Measurement

The double-layer tape was clamped to the middle of the copper tool with high-strength glue, and the adhesion between the PET and GAP pre- and post-plasma treatment was tested by the tensile testing machine. The results are shown in Figure 4. It can be seen from the figure that the tensile stress without the plasma treatment was about 9.84 MPa, as indicated at point A in the figure. After the plasma treatment, the tensile stress was about 22.98 MPa, as indicated by point B in the figure. The tensile stress was increased by about 133.5%. By comparing the surface of the joint after the tooling was pulled apart, it can be found that the GAP and PET were separated thoroughly without the plasma treatment, leaving no residual components. However, after the plasma treatment, the separation of the GAP and PET were not complete enough. There was still a small amount of residue on the surface of the PET. This showed that the adhesion of the PET surface was obviously enhanced after the plasma treatment [33].

**Figure 5:**
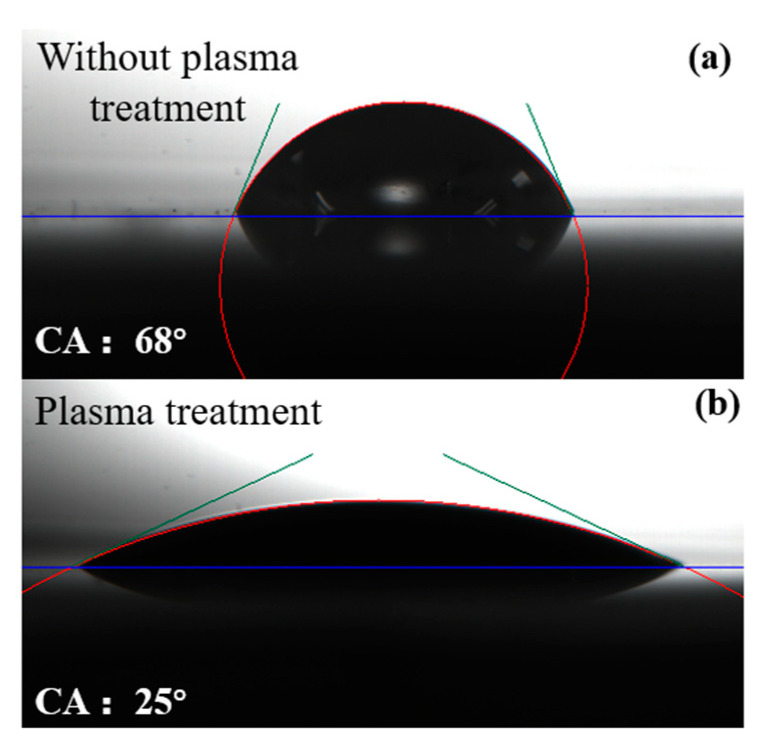
Measurement results of contact angle. (**a**) The contact angle between PET and liquid GAP without plasma treatment, which was about 68°; (**b**) The contact angle between PET and liquid GAP with plasma treatment, which was about 25°.

The contact angle of the liquid GAP and PET was measured by the contact angle measuring instrument. The measurement results are shown in Figure 5. Figure 5a shows the contact angle between the PET and liquid GAP without the plasma treatment, which was about 68°. Figure 5b shows the contact angle of the plasma-treated PET and liquid GAP, which was about 25°. It can be found that the contact angle between the PET and liquid GAP decreased significantly after the plasma treatment. This indicated that the wettability of the PET surface had been significantly improved after the plasma treatment [34,35,36].

#### 3.1.2. Chemical Composition Analysis: XPS Results

**Figure 6:**
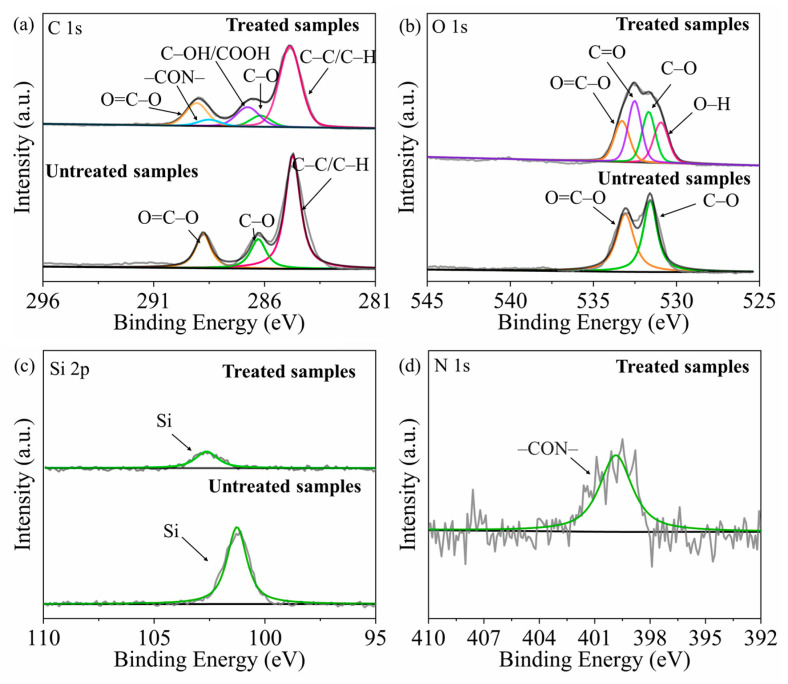
XPS analysis map, including different elements’ bonds pre- and post-plasma treatment: (**a**) C 1s, (**b**) O 1s, (**c**) Si 2p, (**d**) N 1s.

The XPS and AFM were used to characterize the plasma-treated PET surface in order to analyze the mechanism of the enhanced adhesion of the double-layer target band. In order to study the main functional groups introduced into the PET surface after the plasma treatment, high-resolution XPS analysis of various peaks was carried out. The XPS characterization results are shown in Figure 6, and the contents of the various chemical components pre- and post-plasma treatment are shown in Table 1.

As can be seen from Figure 6 and Table 1, the largest change is the N 1s element, whose atomic concentration increases from 0 at% to 1.16 at%. The reason is that after the plasma treatment, a small amount of N was absorbed by the surface of the PET sample in the form of –CON– groups. At the same time, after the plasma treatment, the element content of C 1s decreased, and the element content of O 1s increased. By fitting the C 1s spectra of plasma-treated PET, it can be found that the binding energy peaks correspond to O=C–O (289.00 eV), C–OH/COOH (286.70 eV), C–O (286.12 eV), CON (288.44 eV), and C–C/C–H (284.80 eV), respectively. The changes of the chemical components of C 1s are shown in Table 2. Comparing the C 1s spectra before the plasma treatment, it can be found that a large number of C–OH/COOH bonds appeared after the plasma treatment. This indicated that, after the plasma treatment, the C=O bond broke with the PET surface and formed new bonds. The peaks of C–O and C–C/C–H shifted to the right (about 0.02 eV), and the peaks of O=C-O shifted to the right (about 0.11 eV). This indicated that the electron transfer occurred during the formation of the C–OH/COOH bonds in the sample after the plasma treatment, indicating that the free ions on the PET surface had a change in the valence state. The presence of the C–OH/COOH bond greatly enhanced the adsorption of PET [37].

When fitting the O 1s energy spectrum, it was found that C=O and O–H appeared after the plasma treatment. After the plasma treatment, the C=O bonds as well as the O–H bonds appeared on the sample surface, and the number of oxygen-containing group species increased. Affected by the formation of the new chemical bonds, the peak positions of O=C–O and O–C were slightly shifted. The O–H may originate from water vapor, and O–H formed on the surface of the PET slowly during the plasma treatment. Correspondingly, when the samples were treated with plasma, the CO_2_ in the air dissociated, thus forming C=O bonds. The formation of the two C=O bonds and the O–H bonds enhanced the wettability of the PET surface to varying degrees. In general, the plasma treatment on the PET surface could introduce a large number of free radicals. Moreover, they further promoted the enhancement of the adhesion between the energetic polymers and the PET.

When fitting the Si 2p spectrum, it was found that the concentration of the Si 2p element decreased from 2.77 at% to 0.88 at%. There was no Si element in the PET, but in order to increase the lubrication between the tapes, the factory plated Si on the PET surface. Therefore, after the plasma treatment, the Si element was reduced and the lubrication between the tape was reduced, which further increased the adhesion.

#### 3.1.3. Morphological Analysis: AFM Results

**Figure 7:**
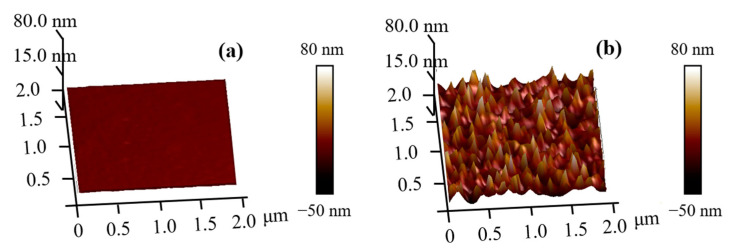
AFM results. (**a**) Untreated sample. The surface roughness of PET without plasma pretreatment was low, and the height difference between the highest and lowest point is about 8.95 nm; (**b**) Treated sample. The surface roughness of PET with plasma pretreatment was high, and the height difference between the highest and lowest point is about 129.85 nm.

The AFM three-dimensional morphology of the PET surface pre- and post-plasma treatment is shown in Figure 7. The surface morphology of the PET was observed by AFM, and the average and root mean square values of the surface roughness were obtained, as shown in Table 3. The average roughness Ra of the PET surface without the plasma treatment was about 1.45 nm, and the root mean square value Rq was about 1.74 nm. The average roughness Ra of the PET surface treated by the plasma was about 14.30 nm, and the root mean square value Rq was about 19.10 nm. It can be seen from the figure that the surface of the PET without the plasma pretreatment was smooth and flat and the surface roughness was low. After the plasma treatment, the surface of the PET became rough and many conical protuberances were observed. This indicated that the surface of the PET was etched due to ion and electron bombardment during the plasma treatment. The increase of surface roughness increased the contact area between the PET and GAP. Therefore, it improved the adhesion performance of the PET and enhanced the binding force of the double-layer tape.

### 3.2. Optimization and Research of Laser Ablation Performance

#### 3.2.1. Comparison of Ablation Performance Pre- and Post-Plasma Treatment

**Figure 8:**
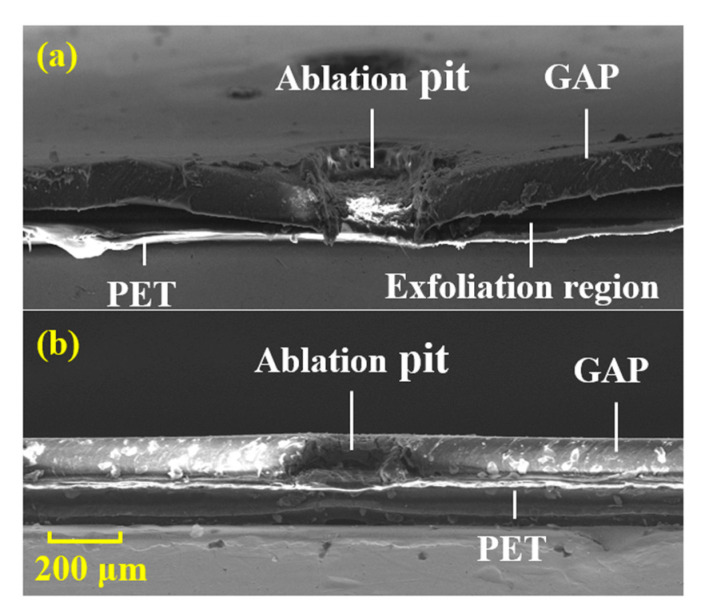
The separation phenomenon of the tape pre- and post-plasma treatment. (**a**) The tape without plasma treatment; (**b**) The tape with plasma treatment.

In order to observe the morphological characteristics of the ablation pits more intuitively, SEM was used to observe the cross-sectional view of ablation pits. Figure 8 shows the ablation pit of the tape and its surrounding tape pre- and post-plasma treatment. The laser pulse width was 200 μs. It can be seen from the figure that the tape without the plasma treatment had a large separation area around the ablation pit. However, there was no separation phenomenon after the plasma treatment. This showed that the adhesion of the tape after the plasma treatment was obviously improved.

**Figure 9:**
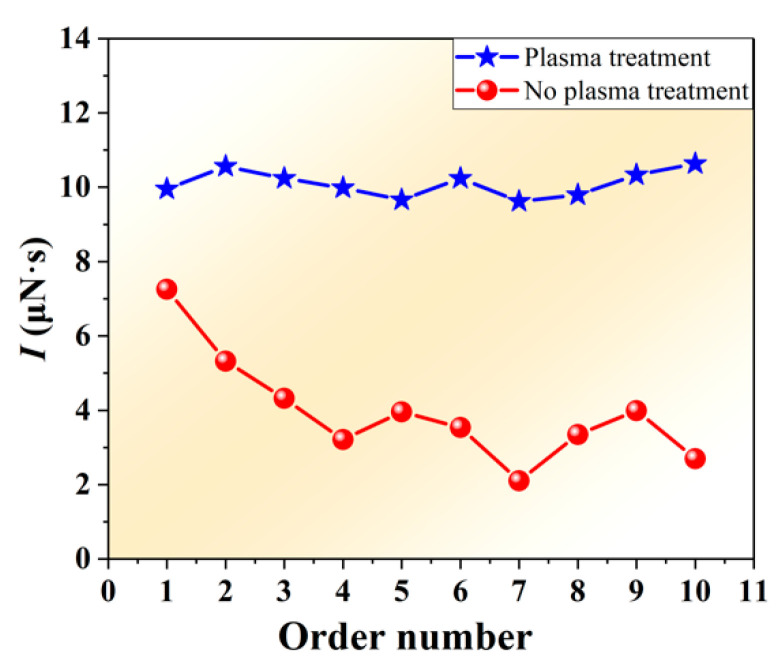
Comparison of *I* of continuous ten ablations. The blue line showed the result of the *I* after plasma treatment. *I* of the tape with plasma treatment was more stable and less volatile. The red line showed the result of the *I* without plasma treatment. The *I* of the tape without plasma treatment decreased obviously and was very unstable.

When the laser pulse width was 200 μs and the ablation pits interval was about 800 μm, the single-pulse impulse (*I*) was continuously measured for 10 times. The results are shown in Figure 9. It can be seen from the figure that the *I* of the tape with the plasma treatment was more stable and less volatile. The *I* of the tape without the plasma treatment decreased obviously and was very unstable. The reason was that the tape without the plasma treatment had poor adhesion, and the GAP and PET were separated during laser ablation. There were two reasons for the decrease of *I* caused by separation of the GAP and PET. One was that the separation of the GAP and PET could cause laser defocus, which greatly reduced the laser power density and the ablation efficiency. Another reason was that, after separation, there was a lack of bottom support during plume spraying, so that the force produced by the laser ablation GAP cannot be fully transferred to the PET. The stability of *I* was the premise to ensure the thrust stability of the laser ablation micro-thruster [38]. In continuous ablation, the separation phenomenon would not only reduce the average thrust, but also cause thrust instability.

#### 3.2.2. Optimization of Ablation Performance

The stability of the double-layer tape after the plasma treatment had been greatly improved. The ablation performance of the double-layer tape treated by plasma was optimized and studied. In this paper, the effect of the laser pulse width on the propulsive performance is compared under the condition that the thickness of the GAP layer is about 180 μm.

**Figure 10:**
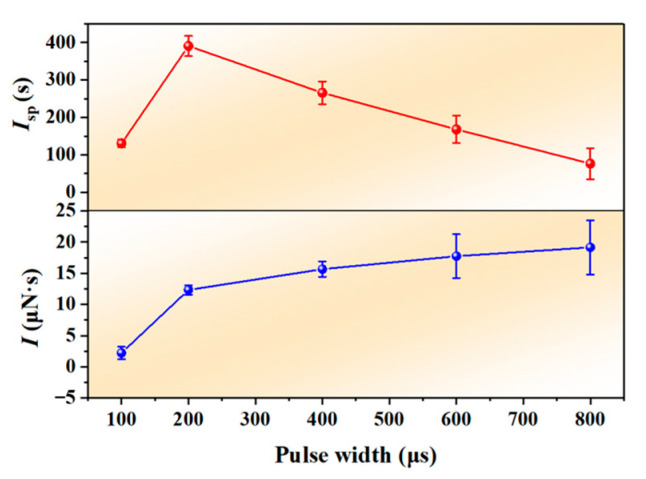
*I* and *I*_sp_ as functions of laser pulse width.

Figure 10 shows the variation curve of *I* and *I*_sp_ with the laser pulse width. It can be seen from Figure 10 that the *I* increased gradually with the increase of the pulse width. When the pulse width was 100 μs, *I* was only 2.22 μN·s, and the *I* was obviously smaller than that of other pulse widths. This was mainly because the GAP was not completely burned through under the condition. When the pulse width was 200 μs, the *I* was 12.33 μN·s. When the pulse width was 800 μs, the *I* reached 19.15 μN·s. However, the change of *I*_sp_ was non-linear, and there was a maximum value at 200 μs, which was 390.65 s. The value of *I*_sp_ at 800 μs pulse width was about 76.25 s. Comparing the numerical results of 800 μs pulse width and 200 μs pulse width, we could find that the *I*_sp_ of the former was about 19.52% of that of the latter, and the *I* increased only 1.55 times.

**Figure 11:**
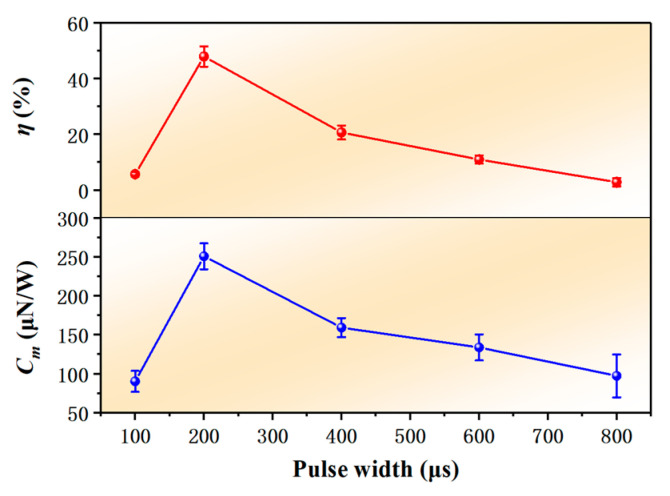
*C*_m_ and *η* as functions of laser pulse width.

Figure 11 shows the curves of *C*_m_ and *η* with laser pulse width. As can be seen from Figure 11, both *C*_m_ and *η* increase first and then decrease. When the pulse width was 200 μs, the maximum *C*_m_ was 250.82 μN/W, and the maximum *η* was 48.01%. At 800 μs pulse width, *C*_m_ and *η* were 97.38 μN/W and 2.88%, respectively. When the pulse width was 800 μs, *C*_m_ and *η* were 38.82% and 6.0% of 200 μs pulse width, respectively.

**Figure 12:**
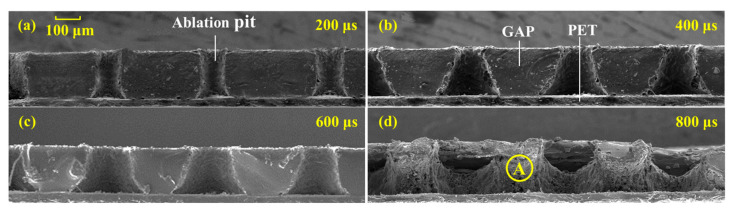
Morphologies of the ablation pits with different pulse widths. (**a**) The sectional view of three consecutive ablation pits with laser pulse width of 200 μs; (**b**) The sectional view of three consecutive ablation pits with laser pulse width of 400 μs; (**c**) The sectional view of three consecutive ablation pits with laser pulse width of 600 μs; (**d**) The sectional view of three consecutive ablation pits with laser pulse width of 800 μs.

In order to further analyze the effect mechanism of the laser pulse width on the propulsion parameters, the ablation pits under different pulse width were observed. Sectional views of the ablation pits at 200, 400, 600, and 800 μs pulse widths are shown in Figure 12. As can be seen from Figure 12, the ablation pits became larger and larger with the increase of the laser pulse width. At the pulse width of 200 μs, the ablation pit was closest to the straight tube. With the increase of the pulse width, the sectional view gradually became trapezoid, especially the influence area at the bottom of the ablation pit became larger and larger. When the pulse width was less than 600 μs, the ablation pits were single. However, when the pulse width was 800 μs, several ablation pits were connected together. The effect of the large pulse width on the bottom of the GAP was greater than that on the top. In addition, from the analysis of the roughness of the ablation pit wall, the ablation pit was “smooth” when the laser pulse width was 200, 400, and 600 μs. When the pulse width of the laser was 800 μs, the ablation pit became coarser, and the small, melted particles were attached to the wall of the ablation pits, as shown in point A in Figure 12d. The intensity distribution function of the laser beam irradiation on the GAP was:

(14) $$I(r)=I_{0}\exp(-r^{2}/\omega^{2})$$

where $I_{0}$ was the effective energy obtained at the bottom of GAP, *r* was the effective radius of the heating area, and ω was the beam diameter with a peak intensity of 1/e^2^. The laser beam had a Gaussian distribution. 

Therefore, the ablation pit morphology at an 800 μs pulse width was the result of the slow heating and melting of the low-energy-density laser beam for a long time. When the laser pulse was large, with the increase of the laser irradiation time, the GAP experienced from ionization to gasification, and then to the melting process. The ablation efficiency and the plume injection efficiency of the gasification and melting processes were very low, resulting in poor propulsion parameters under a large pulse width.

There were three stages of the laser–GAP interaction in the transmitted laser ablation. The first stage was mainly GAP dissociation. The second stage was mainly GAP gasification. The third stage was the GAP first melting, and then gasification. Therefore, in terms of ablation efficiency, the efficiency of the first stage was the highest in the second stage, and the efficiency of the third stage was the lowest. This was also why there were many small, melted particles at the 800 μs pulse width in Figure 12.

Considering the propulsive performance and the ablation pit morphology under different pulse widths, it can be found that the optimal laser pulse width should be the end time of the first stage of the laser ablation. At this time, the shape of the ablation pit was a straight tube, which greatly increased the highest utilization rate of the energetic polymer. Meanwhile, the propulsion parameters, such as *I*_sp_, *C*_m_, and *η*, were also the best.

## 4. Conclusions

In this paper, a method to improve the interface adhesion property of the double-layer tape by plasma treatment was proposed. The XPS and AFM were used to characterize the PET surface pre- and post-plasma treatment, and the mechanism of the enhancement of the adhesion was analyzed. Finally, the ablation performance of the optimized double-layer tape was studied by combining the morphology characteristics of the ablation pit and the propulsion performance.

In the laser ablation propulsion, the interlayer adhesion of the double-layer tape had a great influence on the propulsion performance. The experimental results showed that the interlayer adhesion of the tape treated by the plasma was greatly improved, and its tensile stress increased from 9.84 MPa to 22.98 MPa. The mechanism of the strengthening adhesion was analyzed. First, the plasma treatment to the PET surface can introduce a large number of free radicals. The presence of the C–OH/COOH bond and the O–H bond promoted the enhancement of the adhesion between the energetic polymer and the PET. Second, because of the etching effect of the plasma, the surface roughness of the PET was greatly increased, resulting in the increase of the contact area with the energetic polymer. Every fuel layer with a fixed thickness had an optimal laser pulse width parameter. The laser pulse width could be optimized by combining the morphology of the ablation pit and the propulsion parameters. When the GAP thickness was 180 μm, the optimal pulse width was 200 μs. The optimal values of the specific impulse (*I*_sp_), impulse coupling coefficient (*C*_m_), and ablation efficiency (*η*) were 390.65 s, 250.82 μN/W, and 48.01%, respectively.

## Figures and Tables

**Table 1:** Changes of element content pre- and post-plasma treatment.

Film Surface	PET			
	C 1s at%	O 1s at%	N 1s at%	Si 1s at%
Untreated sample	68.21	28.03	-	2.77
Treated sample	65.47	32.49	1.16	0.88

**Table 2:** Changes of chemical components of C 1s element.

Binding Energy (eV)	Untreated Sample at%	Treated Sample at%	Possible Functional Groups
289.00	19.09	16.25	O=C–O
286.70	-	14.69	C–OH/COOH
286.12	17.15	7.45	C–O
288.44	-	4.77	CON
284.80	63.76	56.84	C–C/C–H

**Table 3:** Surface roughness.

Properties	Ra nm	Rq nm
Untreated sample	1.45 (± 0.22)	1.74 (± 0.39)
Treated sample	14.30 (± 5.86)	19.10 (± 6.34)

## Data Availability

The data presented in this manuscript can be obtained from the corresponding author upon request.
